# Camellia Saponin-Enhanced Sodium Alginate Hydrogels for Sustainable Fruit Preservation

**DOI:** 10.3390/gels11121012

**Published:** 2025-12-16

**Authors:** Lisong Hu, Hongdan Rao, Borong Zhu, Menghao Du, Keqin Xu, Haili Gao

**Affiliations:** 1 Research Institute of Subtropical Forestry, Chinese Academy of Forestry, Daqiao Road 73#, Hangzhou 311400, China; hulisongyls@163.com (L.H.);; 2 College of Food Science and Engineering, Central South University of Forestry and Technology, Shaoshan South Road 498#, Changsha 410004, China; 3 Jiangxi Xinzhongye Tea Industry Technology Co., Ltd., Yanshan 334500, China; 4 Zhejiang Public Welfare Forest and State Forest Farm Management Station, Jianggan District Kaixuan Road 228#, Hangzhou 311000, China

**Keywords:** camellia saponin, hydrogel films, fruit preservation, sodium alginate, sustainable packaging, barrier properties

## Abstract

It is well known that food waste, especially perishable fruits, is one of the pressing issues worldwide, and as much as 50% of harvested fruits are wasted in developing countries as a result of poor preservation methods. Other traditional options such as plastic films or chemical preservatives are harmful to the environment and to our health. In this work, the limitations are overcome through the fabrication of an innovative camellia saponin/sodium alginate (CS/SA) composite hydrogel film that not only recycles agricultural waste but also improves fruit protection. CS/SA films were prepared by ionic crosslinking with CaCl_2_ with different CS content (0–10% *w*/*v*, corresponding to 0–3.1 wt% in air-dried films). Detailed SEM, FTIR, XRD and rheological studies indicated that CS addition led to a gradual microstructural densification, stronger intermolecular interactions (involving hydrogen bonding and electrostatic complexation) and superior viscoelasticity, with the best performance at 8% CS (2.5 wt% in dried film). Mechanical tests confirmed that the stable CS/SA film showed higher tensile strength (152 kPa) and compressive strength (353 kPa) than pure SA (10 kPa) with a relatively low Young’s modulus (0.82 MPa) and high elongation at break (116.33%), which could be easily peeled off from fruit surfaces—an essential benefit of this over stiff chitosan/alginate composites. Structure: The composite film exhibited lower porosity (103.2%), reduced moisture content (94.7%), a controlled swelling ratio (800%) and improved barrier property with a water vapor permeability of 1.3 $\times \;10^{-6}$ g·m^−1^·s^−1^·kPa^−1^ and an oxygen permeability of 1.9 × cm^3^·μm·m^−2^·d^−1^·kPa^−1^. The 8% CS film showed very strong antioxidant activity (86% DPPH scavenging). Results of application tests on bananas and strawberries indicated that the ripening process was delayed by the CS/SA coatings, the decay rate was decreased from 99.9% (uncoated control) to 55.6% after 9 days, the weight loss was reduced to 29.3%, and the fruit’s firmness and titratable acidity were maintained. This degradable, multifunctional hydrogel film has the potential to be a sustainable measure to simultaneously mitigate food waste, valorize agricultural byproducts, and protect the environment, which could offer substantial benefit for enhancing global food security as well as fruit shelf life.

## 1. Introduction

Global food waste poses a significant economic and environmental challenge, with approximately one-third of all food produced annually being lost or discarded [1]. Among perishable commodities, fruits are particularly vulnerable to spoilage. In developing countries, inadequate preservation infrastructure results in postharvest losses reaching up to 50% [2], underscoring the urgent need for effective, accessible preservation technologies that can maintain both safety and quality throughout the supply chain.

Current strategies for extending fruit shelf life during transportation and storage rely predominantly on chemical preservatives and synthetic plastic films [3,4]. However, these conventional approaches present considerable drawbacks. Chemical preservatives raise concerns regarding residue accumulation and potential health impacts, while also contributing to antimicrobial resistance in microorganisms [5,6]. Plastic cling films, though effective at moisture retention, suffer from poor mechanical durability, environmental persistence, and the generation of harmful microplastics [7,8]. Their inadequate protective performance and susceptibility to degradation further limit their utility in practical applications. Consequently, there is a pressing demand for natural, biodegradable, and effective preservation materials that address these limitations while ensuring fruit quality and safety.

Biopolymer-based materials have emerged as promising alternatives for food packaging applications, offering advantages including environmental compatibility, biodegradability, non-toxicity, and sustainability [9,10]. Sodium alginate (SA), a natural polysaccharide comprising poly-β-1,4-mannuronic acid (M units) and α-1,4-L-guluronic acid (G units), has attracted considerable attention due to its biocompatibility, rapid gelation capability, and thermal stability [11]. Despite these favorable properties, the application of SA films in food preservation remains constrained by several critical limitations. Specifically, SA-based films exhibit insufficient mechanical strength, poor elongation at break, and inadequate gas barrier properties [12]. These deficiencies result in brittle films that fracture easily during handling and fail to effectively prevent moisture loss or oxygen ingress—two primary factors contributing to fruit deterioration.

Various strategies have been explored to address these shortcomings, including the incorporation of bioactive compounds such as polyphenols [13], essential oils, proteins, and nanoparticles. While these approaches have demonstrated improvements in antioxidant and antimicrobial activities, they often fail to simultaneously enhance mechanical properties, barrier function, and structural stability. For instance, chitosan/alginate composites can achieve tensile strengths up to 10 MPa, but their excessive rigidity renders the films difficult to remove from fruit surfaces during consumption. Similarly, alginate–pectin films show enhanced flexibility but exhibit poor swelling control (400–500%) and inadequate moisture retention, potentially compromising their effectiveness in maintaining fruit freshness.

Hydrogels, characterized by their hydrophilic three-dimensional network structures, possess inherent capabilities for water absorption, moisture retention, and biocompatibility [14,15]. These properties make them particularly suitable for regulating moisture loss, inhibiting microbial growth, and preserving sensory attributes in food preservation applications [16,17]. However, conventional hydrogels alone typically lack sufficient mechanical strength for durable packaging applications [18]. More critically, existing hydrogel-based preservation films have yet to achieve an optimal balance among mechanical resilience, moderate strength (facilitating easy removal), superior barrier performance, and potent antioxidant activity—all essential attributes for effective fruit preservation.

To address these challenges, this study introduces camellia saponin (CS) as a novel functional modifier for sodium alginate hydrogels. CS is a natural surfactant derived from camellia seeds, representing a significant agricultural byproduct of camellia seed oil production that remains underutilized in sustainable packaging applications. The valorization of CS aligns with circular economy principles, which are gaining increasing prominence in sustainable materials research [19]. Unlike conventional bioactive additives, CS possesses a unique amphiphilic molecular architecture featuring both hydrophobic aglycone moieties and hydrophilic sugar residues. This dual character confers distinct advantages over purely hydrophilic polysaccharides or lipophilic essential oils, underpinning CS’s multifunctional properties including emulsification, antimicrobial activity, and potent antioxidant capacity [14,20,21,22].

The amphiphilic nature of CS enables simultaneous enhancement of multiple alginate hydrogel properties: (1) mechanical reinforcement through strengthened molecular interactions and network densification, (2) improved barrier performance via formation of compact microstructures, (3) robust antioxidant capacity attributed to triterpenoid saponins bearing multiple hydroxyl groups, and (4) moderate mechanical strength permitting easy film removal—a critical practical advantage over existing rigid composite films. Recent advances in polymer–surfactant interactions have demonstrated that incorporating amphiphilic molecules into hydrogel matrices can significantly improve mechanical, barrier, and biological characteristics [23,24]. However, the application of camellia saponin in sodium alginate hydrogels for fruit preservation remains unexplored. Key knowledge gaps persist regarding CS-SA molecular interactions, the influence of CS content on composite hydrogel structure and functionality, and whether these interactions translate to enhanced preservation performance compared to commercial alginate-based systems.

This work systematically investigates CS/SA composite hydrogels as an advanced fruit preservation platform. The study comprehensively examines the following: (1) molecular interactions between CS and SA through spectroscopic and structural analyses, (2) the effects of CS concentration (0–10% *w*/*v*) on microstructure, mechanical properties, swelling behavior, and barrier performance, (3) the antioxidant mechanism of CS within the hydrogel matrix, and (4) the efficacy of CS/SA films in preserving bananas and strawberries relative to pure SA films and uncoated controls. By utilizing agricultural waste from camellia seed oil production, this approach offers a sustainable solution for fruit preservation while simultaneously addressing environmental concerns associated with food waste and packaging materials. The CS/SA composite hydrogel represents a significant advancement in alginate-based packaging, achieving an unprecedented combination of mechanical robustness, superior barrier properties, strong antioxidant activity, and practical applicability. This biodegradable coating technology provides a value-added application for agricultural byproducts consistent with circular economy principles, with substantial implications for enhancing global food security and promoting sustainable development.

## 2. Results and Discussion

### 2.1. Fabrication of Composite Hydrogel Films

For fruit preservation applications, a desirable material should form a film rapidly and exhibit appropriate mechanical properties. Specifically, it should protect fruits during transportation and storage, while being easy to peel off when the fruits are consumed. Conventionally, hydrogel films formed by ionic crosslinking of sodium alginate have poor mechanical properties and tend to be brittle. Therefore, we incorporated amphiphilic camellia saponin to improve the mechanical performance of the single-component sodium alginate hydrogel films.

**Figure 1:**
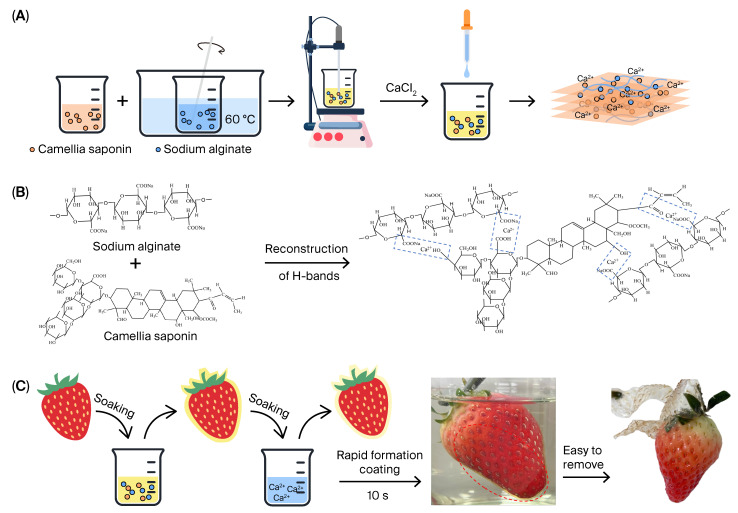
Schematic diagram of the preparation of Camellia saponin and Soudium alginaet (CS/SA) composite hydrogel films (**A**); schematic diagram of the reaction mechanism of CS/SA composite hydrogel (**B**); schematic diagram of fruits coated with hydrogel film (**C**).

The entire preparation process is illustrated in Figure 1A. First, a 3% (*w*/*v*) sodium alginate aqueous solution and camellia saponin aqueous solutions at specific concentrations (0%, 4%, 6%, 8%, and 10% g/mL) were prepared separately. These two solutions were then mixed uniformly. Finally, a 2% (*w*/*v*) calcium chloride aqueous solution was added to trigger the crosslinking reaction: Ca^2+^ cross-linked with α-L-guluronic acid (G units), a component of sodium alginate to yield air-dried CS/SA composite hydrogels with a CS concentration ranging from 1.3 wt% to 3.1 wt%. Meanwhile, the carboxyl and hydroxyl groups of camellia saponin also underwent ionic crosslinking with calcium ions (as shown in Figure 1B). This mechanism aligns with recent findings on cation–alginate complexes, which demonstrate that controlled gelation conditions significantly influence the final hydrogel properties [25].

Additionally, these composite hydrogel films were utilized on fruits, including strawberries and bananas, as illustrated in Figure 1C. The application process started with putting the fruits in a mixture of sodium alginate and camellia saponin for one minute, and then putting them in a calcium chloride solution. After being taken off, hydrogel films formed on the fruit’s surface very quickly (10 s).

### 2.2. Characterization of CS/SA Composite Hydrogels

**Figure 2:**
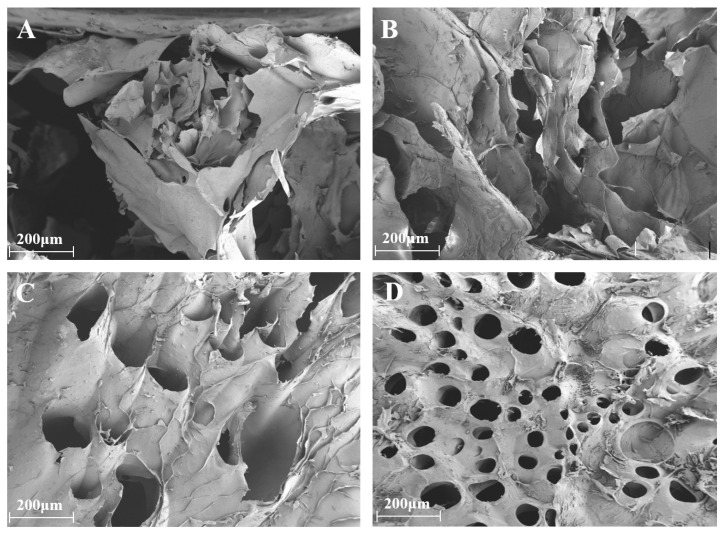
SEM image of pure sodium alginate (SA) hydrogel film (**A**); SEM images of CS/SA composite hydrogels prepared with 4%, 6%, and 8% concentrations of camellia saponin (CS) (**B**–**D**).

SEM analysis showed that the structure of the CS/SA composite hydrogel changed as the CS concentration went up. Pure SA hydrogel exhibited a loose, layered morphology with heterogeneous pores (Figure 2A), aligning with single-network alginate systems [26]. Adding 4%, 6%, and 8% CS (Figure 2B–D) caused densification, which made uniform honeycomb-like structures. This is different from chitosan–alginate systems, which usually only make pore size smaller [27]. The CS/SA system attains enhanced structural uniformity at CS content of 2.5 wt%, indicating that the amphiphilic characteristics of CS molecules promote more efficient network integration compared to solely hydrophilic polysaccharides. Recent research on alginate-based hydrogels underscores the importance of microstructural control in attaining desired barrier and mechanical properties [28].

**Figure 3:**
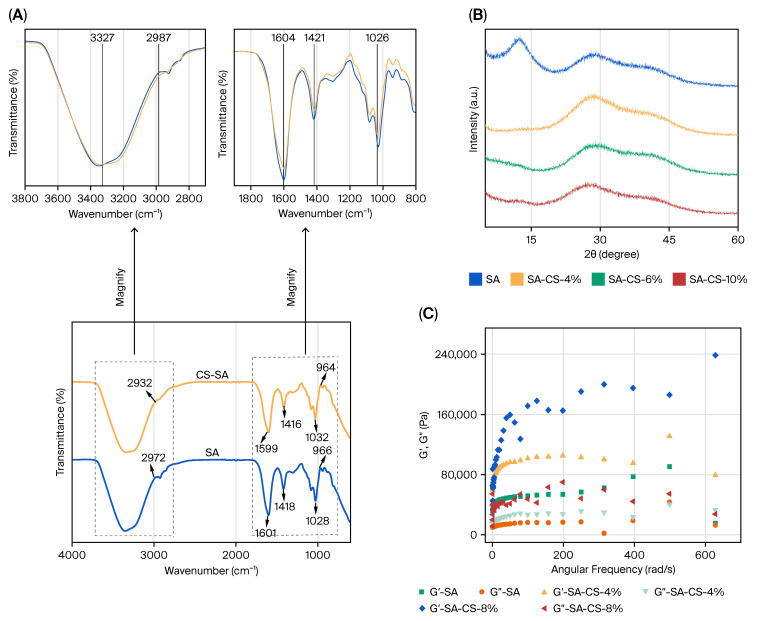
FTIR spectrum of SA and CS/SA composite films (**A**); XRD patterns of pure SA and CS/SA composite films prepared with 8% concentrations of CS (**B**); rheological characterization of pure SA and CS/SA composite films prepared with different concentrations of CS (**C**).

FTIR spectroscopy (Figure 3A) furnished compelling evidence for multi-level molecular interactions. While no dramatic “new peak formation” or “peak disappearance” is observed between pure SA and CS/SA spectra, subtle but meaningful spectral shifts and intensity variations confirm intermolecular interactions (electrostatic attraction and hydrogen bonding) rather than mere physical mixing. The changes in the 1600.92 cm^−1^ and 1417.68 cm^−1^ bands, along with the changes in the -COO stretching vibrations, show that electrostatic interactions between the carboxylate groups, hydroxyl groups of sodium alginate (SA), and Ca^2+^ ions are suggested to be non-trivial. The -OH peak shift from 2931.8 cm^−1^ to 2972.31 cm^−1^ confirms that a large network of hydrogen bonds has formed. These spectroscopic alterations surpass those documented for standard polysaccharide mixtures, indicating that CS’s distinctive molecular architecture, comprising both hydrophobic aglycones and hydrophilic glycosides, facilitates more extensive intermolecular interaction [29,30].

XRD analysis (Figure 3B) showed that adding CS to SA breaks up its semi-crystalline structure while keeping some of its crystallinity, which is important for mechanical flexibility. The appearance of a new peak at $2\theta =13.7^{\circ }$ in 8% CS samples signifies the formation of new ordered domains, in contrast to the complete amorphization commonly seen in synthetic polymer blends [31] regulated alteration of crystallinity corresponds with recent research indicating that moderate disruption of alginate crystallinity improves mechanical properties while maintaining structural integrity [32].

Rheological characterization (Figure 3C) validated solid-like gel behavior, with $G^{\prime }$ consistently surpassing $G^{\prime \prime }$ across all tested frequencies. The progressive rise in both moduli with CS concentration indicates augmented network density and chain entanglement [26]. At an 8% concentration of CS, the storage modulus attained roughly double that of pure SA, signifying substantial network enhancement. This improvement in rheology demonstrates enhanced performance compared to some reported alginate–chitosan systems [28,29], while maintaining complete biodegradability.

### 2.3. Physical Property Enhancement

**Figure 4:**
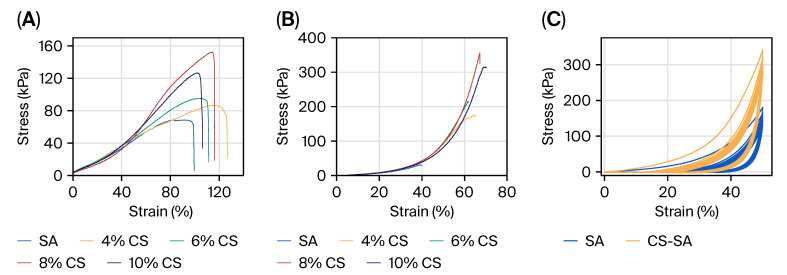
Tensile tests of SA and CS/SA composite films prepared with different concentrations of CS (**A**); compression strength of SA and CS/SA composite films prepared with different concentrations of CS (**B**); cyclic test of SA and CS/SA composite films prepared with different concentrations of CS (**C**).

As shown in Figure 4A,B, compared with the hydrogel films prepared from pure sodium alginate (SA), the Camellia saponin/sodium alginate hydrogel films exhibited superior mechanical properties. Moreover, the mechanical performance of the prepared hydrogel films improved with the increase in Camellia saponin (CS) concentration. When the CS concentration reached 2.5 wt%, the tensile strength of the film could reach 152 kPa; meanwhile, its compressive strength at this concentration was 353 kPa. Compared with pure SA hydrogel (with a compressive strength of only 10 kPa), the CS/SA composite hydrogel film showed better toughness and was less prone to brittleness. Such appropriate, moderate-strength mechanical properties, when compared with chitosan/sodium alginate hydrogel films (which have a tensile strength as high as 10 MPa), not only provide adequate protection for fruits during transportation and storage but also allow for easier peeling when the fruits are consumed [33]. The increased flexibility and cohesive nature of the composite film are important practical benefits. In terms of observations, the CS/SA-2.5 wt% film was cast and dried into a self-supporting film sheet, which could be peeled off easily and did not crumble or crack during handling. This toughness is essential for a coating, as it allows the fruit to be coated completely without developing micro-cracks that would weaken the barrier effect. This is in contrast with brittle films, which may break under the small strain values induced by fruit handling and storage.

Young’s modulus and elongation at break were also determined to further investigate the mechanical property of the film. As summarized in Table 1, the pure SA film had the highest Young’s modulus (1.58 ± 0.12 MPa), and thus the highest stiffness, but the smallest elongation at break (99.6 ± 8.33%), consistent with the film being brittle. The mechanical properties showed a concentration-dependent behavior when CS was added. The CS/SA-1.3% CS film exhibited also a pronounced reduction in Young’s modulus to 0.97 ± 0.11 MPa, as well as a significant increase in elongation at break to 126.9 ± 10.36%, the best result among all the films. This implies that CS is primarily an efficient plasticizer that breaks up the tight packing of alginate polymer chains and results in increased flexibility of the film [34]. An increase in CS concentration to 1.9% led to a slight recovery of rigidity (1.34 ± 0.09 MPa) due to stronger intermolecular forces between CS and SA chains via electrostatic complexation and hydrogen bonding [33], with good ductility (111.4 ± 9.23%).

Notably, the CS/SA-2.5% CS film achieved the optimal balance of mechanical properties, with the lowest Young’s modulus (0.82 ± 0.07 MPa) and high elongation at break (116.33 ± 7.14%). This combination suggests a tough, elastic film that can be bent to a certain extent without break—necessary to conform to uneven surfaces of fruits during application and possibly in storage/transport [35]. The softening of the material is in agreement with our XRD results where a lower crystallinity was observed in the composite films. For higher CS content (3.1%), the modulus slightly increased to 1.09 ± 0.06 MPa, and the elongation decreased to 106.5 ± 8.58%, indicating the beginning of over-crosslinking or phase separation, which negatively affected the flexibility [36]. Its toughness index (the breaking elongation multiplied by the tensile strength) was the highest among all films, which also confirmed its superiority in absorbing energy [37]. Such mechanical properties—intermediate stiffness and high extensibility—are considered very useful for food coating since the film can resist cracking during changes in fruit size due to fruit respiration while providing sufficient strength for handling and storage [38].

Cyclic testing of hydrogels is conducted to evaluate their long-term stability and performance reversibility, so that the hydrogel films can exhibit favorable application performance in fruit preservation applications. As shown in Figure 4C, the testing revealed superior energy dissipation and recovery mechanisms in CS/SA composite hydrogels compared to pure SA. The observed hysteresis loops indicate sacrificial bond rupture, a toughening mechanism recently identified as crucial for high-performance hydrogels [39]. The smaller hysteresis in subsequent cycles for CS/SA samples demonstrates self-healing capability, likely due to reversible hydrogen bonding and hydrophobic interactions introduced by CS molecules. This self-recovery behavior addresses a critical limitation of conventional SA-based materials identified in recent packaging applications [40].

**Figure 5:**
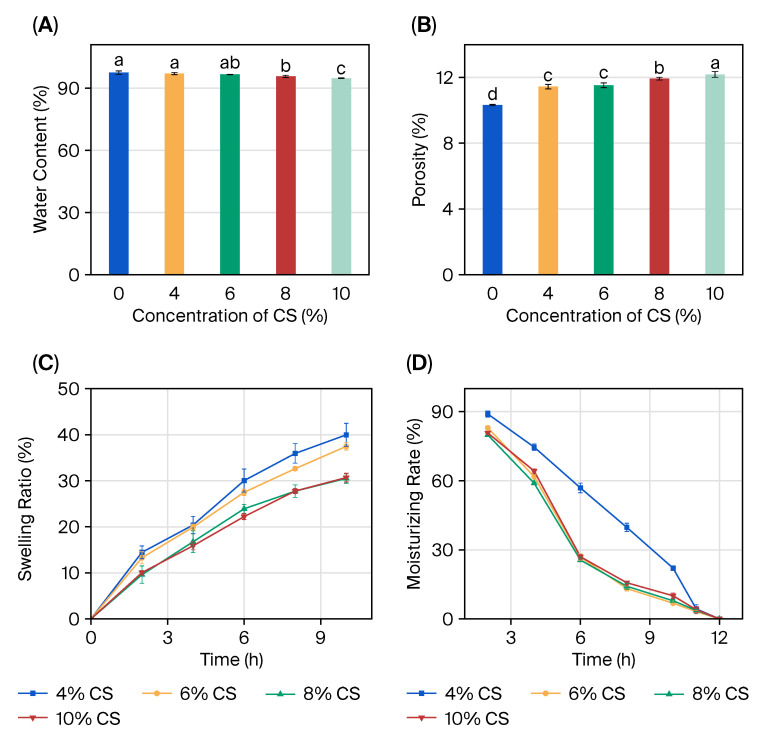
The moisture content (MC) of CS/SA composite films prepared with 0%, 4%, 6%, 8%, and 10% concentrations of CS (**A**); the swelling ratio (SR) of composite films (**B**); the moisturizing rate (MR) of the composite films (**C**); the water solubility (WS) of composite films (**D**). Different letters (a, b, c, d) above the bars indicate statistically significant differences between the groups (*p* < 0.05), as determined by a one-way ANOVA followed by Tukey’s post-hoc test. Groups that do not share a letter are significantly different from one another.

The moisture content of the CS/SA composite hydrogel film went down steadily with the amount of CS (Figure 5A). It went from 97.5% in pure SA to 94.7% in CS/SA composite hydrogels, which shows that the network became denser. This decrease, on the other hand, is not as strong as it is in regular polysaccharide blends. This is probably because CS is amphiphilic and keeps some hydrophilicity. The swelling ratio also went down as the amount of CS went up (Figure 5C), but the 8% CS/SA film kept about 800% of its swelling capacity, which is much higher than the 400–500% which has been reported for recent alginate–pectin films.

The porosity of the prepared SA hydrogels and CS/SA composite hydrogels with different CS concentrations was determined, with the results shown in Figure 5B. Both SA hydrogels and CS/SA composite hydrogels exhibited high porosity values, which were 119.28% and 103.21%, respectively (Table 2). Such high porosity rendered the hydrogel films prone to water absorption and swelling, which was unfavorable for maintaining the stability of the hydrogels’ internal structure. The porosity of CS-incorporated hydrogels was lower than that of SA hydrogels (*p* < 0.05), and the porosity of hydrogels decreased gradually as the CS concentration increased. This phenomenon was attributed to the fact that the addition of CS enhanced the structural compactness of hydrogels, thereby reducing the voids within the CS/SA composite hydrogels. These results indicated that the incorporation of CS could reduce the porosity of CS/SA composite hydrogels, endowing them with a denser network structure. This denser structure made the hydrogels less susceptible to water absorption and swelling, enabling them to block the entry of part of water molecules and oxygen, and thus providing a relatively sealed spatial environment for fruits.

The moisturizing rate profile (Table 3) revealed that the films were capable of maintaining water well. The CS/SA films lost water more slowly at first but held moisture better over time than the pure SA films. When CS was added, the water solubility went up from 34.9% to 63.8% (Table 3). This shows that the molecules interacted better while still being able to break down. The water management features are crucial for food packing since they regulate moisture transfer, preventing both dehydration and condensation.

### 2.4. Barrier Properties and Functional Performance

**Figure 6:**
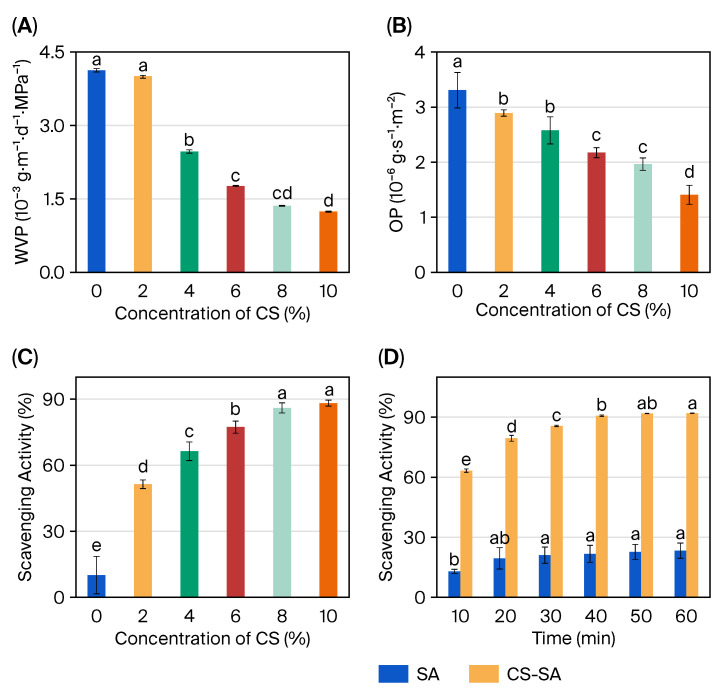
Water vapor permeability (WVP) of CS/SA composite hydrogel films prepared with different concentrations of CS (**A**); oxygen permeability (OP) of CS/SA composite films prepared with different concentrations of CS (**B**); dose-dependent antioxidant activity of CS/SA composite films prepared with 2.5 wt% concentration of CS (**C**); time-dependent antioxidant activity of CS/SA composite hydrogel film prepared with 2.5 wt% concentrations of CS and with 0.0057 ± 0.0002 mm of film thickness (**D**). Different letters (a, b, c, d) above the bars indicate statistically significant differences between the groups (*p* < 0.05), as determined by a one-way ANOVA followed by Tukey’s post-hoc test. Groups that do not share a letter are significantly different from one another.

The water vapor permeability (WVP) and oxygen permeability (OP) of composite films directly affect the freshness of fruits. WVP is a critical parameter for determining whether a film is suitable as a packaging material; films with low WVP can protect food from moisture damage [41]. As shown in Figure 6A and Table 4), the SA hydrogel composite film had a loose structure, allowing water molecules to pass through relatively easily, resulting in a relatively high water vapor transmission rate of 4.1 × 10^−6^g^−1^·m^−1^·s^−1^·kPa^−1^. The incorporation of CS reduced the gaps in the composite film, and the CS/SA composite hydrogel film formed a dense microstructure that prevented water molecule penetration and reduced the water vapor transmission rate. Additionally, the water vapor permeability of the composite film decreases with increasing CS concentration. At a CS concentration of 8%, the WVP of the CS/SA composite hydrogel film is 1.3 × 10^−6^g^−1^·m^−1^·s^−1^·kPa^−1^. This finding is consistent with the results reported by Zhang et al. [42], indicating that CS/SA composite hydrogel films have potential as fruit preservation materials. To reduce the exchange rate of moisture and oxygen between food and the environment, low WVP and OP are generally required to prevent oxidation and deterioration. Figure 6B showed the oxygen permeability of the composite films. It was evident that the SA film had the highest oxygen transmission rate of 3.3 × cm^3^·μm·m^−2^·d^−1^·kPa^−1^, partly due to its less compact structure compared to CS/SA films. The CS/SA composite hydrogel film exhibits an oxygen transmission rate of 1.9 × cm^3^·μm·m^−2^·d^−1^·kPa^−1^. Owing to strong physical interactions (electrostatic attraction and hydrogen bonding), the CS/SA film is more compact than the pure SA film, forming a uniform and dense barrier that hinders the diffusion of water and oxygen molecules [43]. These results demonstrate that the CS/SA film structure can block the entry of part of oxygen and water vapor from the air.

The DPPH free radical scavenging assay was used to determine the antioxidant capacity of the films in ethanol, which represents the “maximum theoretical antioxidant capacity”. Oxidation typically causes fruit discoloration; it destroys nutrients (e.g., vitamin C and carotenoids) and severely impairs the appearance and taste of food products [47]. This study investigated whether camellia saponin, when coated in sodium alginate as an active agent, could retain its antioxidant activity. As shown in Figure 6C,D, the antioxidant activity of all hydrogel films exhibited a dose-dependent relationship with increasing antioxidant content. Specifically, the DPPH free radical scavenging capacity of the composite films was gradually enhanced as the CS concentration increased. The pure SA film showed a scavenging activity of 10.1%, while the CS/SA composite hydrogel film with 8% CS added achieved a radical scavenging activity of 86%. The enhanced antioxidant capacity was attributed to the presence of triterpenoid saponins and hydrogen atoms in CS, as well as hydroxyl and triglyceride groups in the triterpene structure, along with small hydrophobic molecules. These components provide the compound with more oxygen atoms and electrons to bind with free radicals, and also facilitate its absorption and distribution in tissues [48]. Furthermore, the radical scavenging activity of the CS/SA composite hydrogel film with 8% CS was measured at different time points. The results indicated that the composite film exhibited time-dependent scavenging activity. Notably, the DPPH free radical scavenging capacity of the CS/SA composite hydrogel film was approximately eight times that of the SA film, demonstrating that CS can improve the antioxidant capacity of the film and help inhibit the oxidation of packaged food.

### 2.5. Practical Application in Fruit Preservation

**Figure 7:**
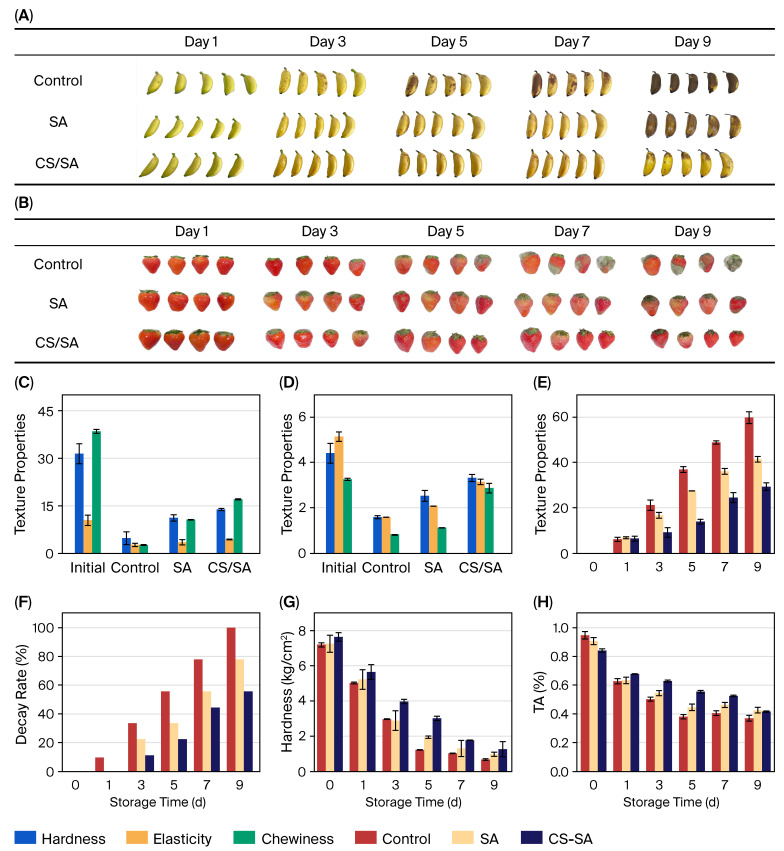
Determination of the quality of fruits. Photos of changes in the appearance of bananas during storage (**A**); photos of changes in the appearance of strawberries during storage (**B**); textural properties of banana with skin (**C**); textural properties of banana without skin (**D**); changes in weight of strawberries during storage (**E**); decay rate of strawberries during storage (**F**); changes in hardness of strawberries during storage (**G**); changes in titratable acid (TA) content of strawberries during storage (**H**).

Figure 7A shows that bananas that were coated with SA hydrogel films and CS/SA composite hydrogel films stayed better on the surface than bananas that were not coated. This shows that the coating treatment did help to keep the fruit fresh. The SA hydrogel films on the banana surface cracked while the CS/SA-coated bananas stayed mostly whole, with only a small part of the film cracking at the end of storage. This was because SA hydrogel films are brittle, do not stretch well, and have bad mechanical properties. On the other hand, CS/SA composite hydrogel films have a denser network structure and better extensibility, which is in line with the previous results for mechanical properties. These results showed that adding CS can improve the mechanical properties of hydrogel films, which will help them work better for preserving fruit. We kept bananas that had been treated with hydrogel films at room temperature for a week. Then, we used a texture analyzer to measure their hardness, surface elasticity, and chewiness. You can see the results in Figure 7C,D. Uncoated bananas were less hard, elastic, and chewy than coated ones. This shows that both SA and CS/SA coating treatments slowed down the ripening of bananas and kept them from becoming soft and spoiling. Bananas coated with CS/SA composite hydrogel films had higher values for all of the above texture parameters than bananas coated with SA films. Adding CS makes the hydrogel film structure denser, which makes it better at sealing the banana surface. Better air isolation leads to better preservation.

As shown in Figure 7B, the strawberries that were kept for 9 days in CS/SA composite hydrogel films did not show any clear signs of rot, kept their good color, and stayed moist enough. The control group, on the other hand, was made up of strawberries from the same batch that did not have any film coating. These strawberries started to mold on day 3, and the mold became worse as time went on. Figure 7F showed that the control group’s decay rate went up quickly during storage, which caused the food to spoil quickly. The CS/SA film group, on the other hand, did not rot at all in the first three days, and its decay rate only went up slowly after that, staying low throughout. The CS/SA composite film group had a decay rate of 55.6% on day 9, which was much lower than the SA group’s rate of 77.8%. The control group, on the other hand, had a rate of 99.9% and was basically no longer edible.

In addition to rot and visual quality, weight loss, another key sign of fresh strawberry preservation, also varied a lot between the three treatment groups (as shown in Figure 7E). As storage time increased, all groups exhibited varying levels of weight loss: after 9 days, the CS/SA film-coated group demonstrated the lowest weight loss rate at 29.3%, while the uncoated control group reached 59.7%. The significant difference arises from the dense network structure of the CS/SA composite hydrogel film, which effectively diminishes surface moisture evaporation and obstructs water vapor exchange with the external environment—addressing the primary factor contributing to weight loss in fresh fruits during storage.

Strawberry hardness, an important measure of textural quality, went down steadily over time in all three groups, going from 7.6 kg/m^2^ to 1.2 kg/m^2^ (see Figure 7G). This decrease in hardness is a normal part of fruit ripening that happens when the cell walls break down and the starch breaks down, making the fruit softer. The CS/SA film-coated group had significantly higher hardness in the middle and late storage stages than the control group and the SA film-coated group. This further supports the idea that the coatings slow down ripening.

Along with texture, the titratable acid content, which is a key measure of strawberry flavor and freshness, also went down in all groups as storage time went on, going from 0.94% to 0.41% (as shown in Figure 7H). Titratable acid is closely related to how fruits breathe; its levels go down mostly because organic acids are used up during respiration. The CS/SA film-coated group exhibited a slower rate of titratable acid decline in comparison to the SA group and the uncoated control group. This is because the coating can stop too much breathing by blocking oxygen from entering. This slows down the breakdown of organic acids and better maintains the strawberries’ original flavor and nutritional value. This is consistent with the coating’s overall effect of delaying ripening, as seen in tests for rot, weight loss, and hardness.

### 2.6. Scalability and Industrial Implementation Considerations

Although CS/SA composite hydrogel films possess desirable characteristics for fruit preservation, some problems still need to be overcome for their application at the industrial scale. As a natural byproduct of camellia seed oil production, camellia saponin is well known to be cost-effective compared to its synthetic counterparts. However, when the supply chain and scalability are considered, they can conflict with each other in the case of mass production. The present study was performed with CS having 95% purity; however, an economic evaluation will be necessary for large-scale production to assess the cost of sourcing CS compared with that of other natural preservatives. Formation of CS/SA film is a complex process, which includes mixing of SA and CS solutions, adding the controlled amounts of CaCl_2_, and gelation/solidification—all steps have to be well controlled. Major parameters and the associated challenges are summarized as follows: (1) The current lab-scale solution casting procedure (employing molds that are 2 mm deep) may not be directly applicable to industrial spray or dip coating methods. Variation in the depth of coatings may have an impact on the barrier and mechanical properties of the resulting bionanocomposite film. (2) The film gelation (10 s) in a subsecond range for fruit coating is beneficial; however, large-scale commercial production will require machine methods to uniformly achieve the Ca^2+^-mediated crosslinking of multiple units of fruits. (3) Preliminary results suggest that CS/SA films maintain their integrity under laboratory storage conditions (4 °C, 50% RH); however, additional testing is necessary under the following conditions to confirm the applications of these films in real-world scenarios.

- Temperature dependence: The behavior of the film at elevated temperatures (e.g., 25–35 °C, along the supply chain pathway in the tropical application environment).
- Effect of humidity: Variations in water vapor sorption and mechanical properties of the film under different humidity conditions (30 to 90% RH).
- Stability for dried film products: Stability of films at the pre-application stage, particularly the long-term stability of dried films stored prior to use for more than 6 months.

## 3. Conclusions

In this work, a novel camellia saponin/sodium alginate (CS/SA) composite hydrogel film was successfully fabricated to overcome the bottlenecks of traditional biopolymer-based food packaging materials and add value to agricultural waste. By performing a comprehensive study on the influence of CS concentration (0–3.1 wt%) on hydrogel morphology and performance, we demonstrated that the amphiphilic property of CS allows for the simultaneous improvement of several properties required for effective fruit preservation.

A gradual densification of the microstructure was observed with the addition of CS, leading to a transformation of the loose and heterogeneous pore configuration of neat SA into homogeneous honeycomb-like structures. The results of FTIR and XRD analyses revealed increased intermolecular interactions via hydrogen bonding and electrostatic complexation, and regulated crystallinity disruption that retained the structural integrity while enhancing the ductility. Rheological analysis revealed that 8% CS led to a twofold increase in the storage modulus in comparison with pure SA, denoting the existence of a strong network within the hydrogel.

The CS/SA-2.5 wt% film exhibited a good balance of reasonable strength (tensile: 152 kPa; compressive: 353 kPa), low Young’s modulus (0.82 MPa), and excellent elongation at break (116.33%). This enhanced mechanical characteristic, which is 35 times stronger than pure SA yet orders of magnitude more flexible than rigid chitosan/alginate sheets (10 MPa), allows the film to shield fruits during processing and handling while being peeled off effortlessly at the point of consumption, thus overcoming a significant practical barrier for current composite films. Considerable enhancement in barrier properties (WVP: 1.3 × 10^−6^g^−1^·m^−1^·s^−1^·kPa^−1^; OP: 1.9 × cm^3^·μm·m^−2^·d^−1^·kPa^−1^) and controlled swelling behavior (800%, much higher compared to reported alginate–pectin films at 400–500%) were observed for CS/SA films along with strong antioxidant properties (86% DPPH scavenging at 2.5 wt% CS). Properties such as these work synergistically for moisture retention and oxidative protection.

Coating work on bananas and strawberries proved that CS/SA coatings prolong shelf life by retarding ripening and decay (decay rates reduced from 99.9% in uncoated controls to 55.6% after 9 days), reducing fruit weight loss to 29.3%, and maintaining firmness and titratable acidity. These results confirm the practical viability of CS/SA films for fruit preservation. The conversion of camellia saponin, a major waste product of camellia seed oil manufacture, into a value-added functional material in this work represents an effective agricultural waste valorization approach complementing the circular economy concept. The biodegradable and non-toxic coating could be a green alternative to traditional plastic films and chemical preservatives.

The high efficiency of CS/SA composites is attributed to the unique amphiphilic property of CS, which can (1) exert plasticizing effect to improve flexibility; (2) result in compact microstructures to enhance barrier properties; (3) possess multiple hydroxyl groups accounting for antioxidant potential; and (4) involve reversible hydrogen bonding to enable self-healing capacity. This multifunctional improvement makes CS different from traditional hydrophilic polysaccharides or lipophilic essential oils.

Despite providing the basis for understanding the properties and fruit protection efficacy of CS/SA hydrogels, several aspects warrant future investigation: (1) long-term shelf life studies under different storage conditions; (2) scaling up and industrialization; (3) application to other fruit varieties and climacteric/non-climacteric fruit species; (4) detailed antimicrobial mechanism and spectrum; (5) life cycle assessment and techno-economic analysis; and (6) inclusion of additional bioactive materials for functionality enhancement.

In summary, the CS/SA hybrid hydrogel represents a new paradigm in biopolymer-based food packaging, realizing an unprecedented level of mechanical resilience, barrier performance, antioxidant activity, and real-life applicability. This study not only offers a green solution for fruit preservation but also demonstrates the general feasibility of agricultural residue valorization in the production of advanced functional materials. By addressing the synergistic challenges of food waste, environmental pollution, and resource sustainability, this edible coating technology has significant implications for enhancing global food security and sustainable development.

## 4. Materials and Methods

### 4.1. Materials

Sodium alginate (SA) was purchased from Shanghai Meclin Biochemical Technology Co., Ltd., Shanghai, China. Camellia saponin (CS) powder (95% purity, GB/T 41549-2022) was obtained from Jiangxi Xinzhongye Tea Industry Technology Co., Ltd. Calcium chloride (${\mathrm{CaCl}}_{2}$) was sourced from China National Pharmaceutical Group Chemical Reagent Co., Ltd., Shanghai, China. N,N’-Methylenebisacrylamide (MBA) was obtained from Shanghai Aladdin Reagent Co., Ltd., Shanghai, China., and 1-diphenyl-2-picrylhydrazyl (DPPH) was obtained from Shanghai Macklin Biochemical Technology Co., Ltd., Shanghai, China. All reagents were of analytical grade, and all solutions were prepared using deionized water.

### 4.2. Preparation of Hydrogel Films

#### 4.2.1. Fabrication of Sodium Alginate (SA) Hydrogel Film

Sodium alginate powder (3%, *w*/*v*) was dissolved in distilled water at 60 °C under magnetic stirring (300 rpm) for 2 h to accelerate complete dissolution and ensure solution homogeneity. After complete dissolution, the solution was cooled to room temperature, before adding Camellia saponin to avoid thermal degradation of bioactive compounds, followed by storage of the solution at 4 °C for further use. Subsequently, a 2 wt% CaCl_2_ aqueous solution was added dropwise to the SA aqueous solution under constant stirring (magnetic stirrer, 1000 rpm) for 4 h to obtain the SA hydrogel film-forming solution.

Next, 25.0 mL of this solution was cast onto a 120 mm × 120 mm acrylic mold with a 2 mm depth and degassed overnight at room temperature. The resulting films were stored at 4 °C and 50% relative humidity for future analysis.

#### 4.2.2. Fabrication of Camellia Saponin/Sodium Alginate (CS/SA) Hydrogel Films

CS aqueous solutions were prepared by dissolving 0, 4, 6, 8, or 10 g of CS in 100 mL of deionized water at 25 °C, with constant stirring (magnetic stirrer, 2000 rpm) for 2 h. Through this procedure, CS aqueous solutions with concentrations of 2%, 4%, 6%, 8%, and 10% (m/v) were successfully obtained. The SA aqueous solution was prepared according to the procedure described above.

For the CS/SA composite hydrogel film-forming solutions, the CS and SA aqueous solutions were mixed at a 1:2 ratio with constant stirring (magnetic stirrer, 1000 rpm) for 1 h and then stored at 4 °C. Subsequently, a 2% CaCl_2_ aqueous solution was added dropwise over 4 h, and the mixture was stored at 4 °C for further use.

Next, 25.0 mL of the CS/SA composite hydrogel film-forming solution was cast onto a 120 mm × 120 mm acrylic mold with a 2 mm depth and then degassed overnight at room temperature. The resulting films were air-dried naturally with 0–3.1 wt% of CS (Table 5) before being stored at 4 °C and 50% relative humidity for future analysis.

### 4.3. Characterization of CS/SA Composite Hydrogel Films

- FTIR: Lyophilized samples were analyzed (4000–500 cm^−1^, 4 cm^−1^ resolution) using a Nicolet iS50 FTIR spectrometer (Thermo Fisher Scientific, Waltham, MA, USA) with the KBr pellet method.
- SEM: Lyophilized CS/SA composite films were fractured in liquid nitrogen, gold-coated, and imaged at 200× magnification using a JSM-6390LV SEM (JEOL Ltd., Tokyo, Japan).
- XRD: Patterns were recorded from 5 to 40 ° (2 *θ*) at 0.05 rad/s, using a D8 Advance diffractometer (Bruker, Karlsruhe, Germany) at 40 kV and 30 mA.
- Rheology: Storage ($G^{\prime }$) and loss ($G^{\prime \prime }$) moduli were measured at 25 °C, using a Discovery HR-2 rheometer (TA Instruments, Milford, MA, USA), with frequency sweeps from 0.1 to 10 Hz at 0.1% strain.

### 4.4. Mechanical Property Assessment

According to previously reported methods [49], hydrogel films were cut into dumbbell-shaped specimens (width 4 mm and length 25 mm) and tested using a universal testing machine Instron 5967, (Instron Corp., Boston, MA, USA) with a 20 N load cell at 100 mm/min for tensile strength, compression, and cyclic compression. Stress–strain curves were generated, measurement was performed on three samples, and the results are presented as averaged ± SD.

### 4.5. Moisture Content and Swelling

According to the methods of [50] with minor modifications, the prepared hydrogel films (4 cm × 4 cm) were initially weighed (*m*), dried at 105 °C for 12 h ($m_{1}$), rehydrated in water for 24 h ($m_{2}$), and then dried again to constant weight ($m_{3}$). The properties were calculated as follows:

(1) $$\mathrm{Moisture}\;\mathrm{Content}\;(\%)=\frac{m-m_{1}}{m}\times 100$$

(2) $$\mathrm{Swelling}\;\mathrm{Ratio}\;(\%)=\frac{m_{2}-m_{1}}{m_{1}}\times 100$$

(3) $$\mathrm{Water}\;\mathrm{Retention}\;\mathrm{Rate}\;(\%)=\frac{m_{3}-m_{1}}{m_{2}-m_{1}}\times 100$$

(4) $$\mathrm{Water}\;\mathrm{Solubility}\;(\%)=\frac{m_{1}-m_{3}}{m_{1}}\times 100$$

### 4.6. Porosity of the SA and CS/SA Films

The porosity of the dried SA and CS/SA films was measured according to a reported reference with minor modifications [51]. The porosity of the samples was calculated as follows:

(5) $$\mathrm{Porosity}\;(\%)=\frac{m_{2}-m_{1}}{\rho \times V}\times 100$$

where $m_{1}$ is the mass of dry hydrogel, $m_{2}$ is the mass of ethanol-saturated hydrogel, ρ is the density of ethanol (0.789 g·cm^−3^), and V is the geometric volume of the hydrogel sample.

### 4.7. Thickness Measurement

According to [52], a digital thickness gauge XHA217 (Mitutoyo Corp., Kawasaki, Japan) with a precision of 0.001 mm was used to measure the film thickness. At several points on each sample, measurements were taken and the average was found.

### 4.8. Antioxidant Activity

The antioxidant properties of the specimens were examined following known techniques in the literature [53]. Initially, 25 mg of CS/SA films was weighed and dispersed in 2 mL of ethanol with stirring for 30 min. The dispersion was centrifuged at 3000 rpm for 5 min, and the clear supernatant was collected for analysis to minimize light scattering interference. Subsequently, 1 mL of the supernatant was mixed with 2 mL of an ethanol-based DPPH solution (2,2-diphenyl-1-picrylhydrazyl at 25 mg/L). Absorbance was then measured at 517 nm under room temperature conditions with a UV-Vis spectrophotometer. Antioxidant activity was evaluated in two modes: dose-dependent, where films with CS concentrations of 0%, 2%, 4%, 6%, 8%, and 10% were tested for 30 min; and time-dependent, where films with the optimal CS concentration (identified from the dose-dependent results for maximum scavenging activity) were assessed at intervals of 10, 20, 30, 40, 50, and 60 min. Scavenging activity was calculated as follows:

(6) $$\mathrm{DPPH}\;\mathrm{radical}\;\mathrm{scavenging}\;\mathrm{activity}\;(\%)=\frac{A_{b}-A_{s}}{A_{b}}\times 100$$

where $A_{s}$ represents the absorbance of the samples and $A_{b}$ represents the absorption of the control solution at a wavelength of 517 nm.

### 4.9. Barrier Properties

#### 4.9.1. Water Vapor Permeability (WVP)

The water vapor permeability (WVP) was assessed following the methodology of [54] with certain changes. Each film was sealed over a permeable cup containing 30 g of anhydrous silica gel (0% relative humidity). The cup was thereafter positioned in a desiccator filled with distilled water, and its weight was recorded every 24 h for a duration of 7 days. The WVP was determined from the weight increase of the cup as follows:

(7) $$\mathrm{WVP}=\frac{\Delta m\times d}{s\times t\times p}$$

where Δm denotes the weight variation of the bottle prior to and subsequent to the test, *d* represents the thickness of the composite films, *s* indicates the water vapor-permeable area of the films, *t* signifies the duration of the test, and *p* is the differential water vapor pressure across the two sides of the composite films, approximately 10 kPa under the specified test conditions.

#### 4.9.2. Oxygen Permeability (OP)

The oxygen permeability was assessed following the methodology outlined in the prior study [55]. In summary, 3 g of a deoxidizing agent, consisting of reduced iron powder, sodium chloride, and activated carbon in a 0.5:1.5:1 ratio, was positioned at the base of a weighing bottle and subsequently covered with hydrogel film samples. The original mass of the weighing bottle was documented. Thereafter, the bottles were positioned in a desiccator with a saturated barium chloride solution at the base (maintaining 90% relative humidity) and incubated for 48 h at 25 °C. The oxygen permeability (OP) of the active film was determined as follows:

(8) $$\mathrm{OP}=\frac{m_{f}-m_{i}}{t\times A}$$

where $m_{f}$ denotes the final weight of the weighing bottle after a 48 h equilibration, $m_{i}$ represents the beginning weight of the weighing bottle, *t* indicates the duration of equilibration, and *A* signifies the area of the exposed film surface.

### 4.10. Fruit Coating Test

Fresh bananas and strawberries were purchased from the same supplier in a single batch, selected for uniform maturity, size, and absence of defects, and then randomly assigned to three treatment groups: control (uncoated), SA film-coated, and CS/SA film-coated (optimal CS concentration). Each group consisted of three biological replicates containing 15 bananas or 25–30 strawberries per replicate.

For SA film-coated groups, fruits were dipped in SA solution for 30 s, drop-dried for 2 min, and sprayed with 2% CaCl_2_ for 10 s. For CS/SA film-coated groups, fruits were immersed for 30 s, drip-drained for 2 min, sprayed with 2% CaCl_2_ for 10 s, and air-dried for 30 min. Control groups received no treatment.

All groups were stored in an environmental chamber at 25 °C and 40% relative humidity without forced convection. Fruits were placed on perforated polypropylene trays with ∼5 cm spacing to promote airflow and prevent moisture accumulation. Trays were randomly positioned within the chamber, and the door was opened briefly daily for measurements and air exchange.

During storage, fruit appearance (visual scoring on a 1–5 scale) and hardness (Texture Analyzer, Stable Micro Systems, Surrey, UK) were assessed daily. Three fruits per replicate were randomly selected for each measurement, with the final data reported as the mean ± SD of three biological replicates.

### 4.11. Statistical Analysis

Experiments were conducted in triplicate, with data reported as mean ± SD. One-way ANOVA (SPSS 27.0, *p* < 0.05) and Duncan’s test were used to evaluate significance. Graphs were plotted using Origin 2021.

## Figures and Tables

**Table 1:** Mechanical properties of SA and CS/SA composite films with different initial CS solution concentrations.

Initial CS Solution Concentration (% *w*/*v*)	Young’s Modulus (MPa)	Elongation at Break (%)
SA	1.58 ± 0.12	99.6 ± 8.33
CS/SA-1.3% CS	0.97 ± 0.11	126.9 ± 10.36
CS/SA-1.9% CS	1.34 ± 0.09	111.4 ± 9.23
CS/SA-2.5% CS	0.82 ± 0.07	116.33 ± 7.14
CS/SA-3.1% CS	1.09 ± 0.06	106.53 ± 8.58

**Table 2:** The thickness SA and CS/SA composite films.

Hydrogel Type	CS Concentration (%)	Film Thickness (mm)
SA	0	0.063 ± 0.004
CS/SA-1.3% CS	4	0.062 ± 0.006
CS/SA-1.9% CS	6	0.060 ± 0.003
CS/SA-2.5% CS	8	0.057 ± 0.002
CS/SA-3.1% CS	10	0.056 ± 0.005

**Table 3:** The solubility, moisture content and moisture rate of SA and CS/SA composite films.

Hydrogel Type	Water Solubility (%)	Moisturizing Rate (%)	Moisture Content (%)
SA	34.92 ± 0.07	0.8898 ± 0.013	0.9747 ± 0.008
CS/SA	63.89 ± 0.02	0.7991 ± 0.004	0.9475 ± 0.002

**Table 4:** Comparison of water vapor permeability (WVP) and oxygen permeability (OP) of the spray-coated CS/SA film with other polysaccharide-based films reported in the literature.

Film Material	Method	WVP	OP	Ref.
		**(10^−6^g^−1^·m^−1^·s^−1^·kPa^−1^)**	**(cm^3^·μm·m^−2^·d^−1^·kPa^−1^)**	
CS/SA (This Work)	Spray	1.30 ± 0.15	1.9 ± 0.25	–
Pure Sodium Alginate	Spray	4.10 ± 0.14	3.30 ± 0.31	–
Pure Sodium Alginate	Casting	2.65 ± 0.12	5.20 ± 0.45	[44]
Pure Chitosan	Casting	1.92 ± 0.08	4.80 ± 0.30	[45]
CMC	Casting	1.54 ± 0.05	12.50 ± 1.20	[46]

Note: WVP and OP values from literature were converted to the unit used in this study for direct comparison.

**Table 5:** CS content in air-dried natural hydrogel films.

Initial CS Concentration (*w*/*v*)	Mass of CS (g in 100 mL)	wt% of CS in Air-Dried Film
0%	0	0%
4%	4	1.3%
6%	6	1.9%
8%	8	2.5%
10%	10	3.1%

## Data Availability

The original contributions presented in this study are included in the article. Further inquiries can be directed to the corresponding authors.
